# Complete Genome Sequence of *Arcanobacterium* sp. Strain 2701, Isolated from a Harbor Seal

**DOI:** 10.1128/MRA.00652-20

**Published:** 2020-09-17

**Authors:** Maria Borowiak, Mazen Alssahen, Abdulwahed Ahmed Hassan, Christoph Lämmler, Osama Sammra, Burkhard Malorny, Laura Uelze, Antonia Kreitlow, Ellen Prenger-Berninghoff, Ursula Siebert, Madeleine Plötz, Amir Abdulmawjood

**Affiliations:** a German Federal Institute for Risk Assessment (BfR), Department for Biological Safety, Berlin, Germany; b Institut für Hygiene und Infektionskrankheiten der Tiere, Justus-Liebig-Universität Gießen, Gießen, Germany; c Institute of Food Quality and Food Safety, Research Center for Emerging Infections and Zoonoses (RIZ), University of Veterinary Medicine Hannover, Hannover, Germany; d Institute for Terrestrial and Aquatic Wildlife Research (ITAW), University of Veterinary Medicine Hannover, Hannover, Germany; University of Maryland School of Medicine

## Abstract

*Arcanobacterium* spp. are Gram-positive bacteria which can be found in a wide range of hosts and can be associated with disease in humans and animals. Here, we announce the complete genome sequence of *Arcanobacterium* sp. strain 2701, isolated from a harbor seal from the North Sea.

## ANNOUNCEMENT

The genus *Arcanobacterium* is comprised of 10 classified species (https://www.ncbi.nlm.nih.gov/Taxonomy/Browser/wwwtax.cgi?id=28263) of facultative anaerobic, asporogenous Gram-positive bacteria found in a variety of hosts, including seals (1–3), cats and dogs (4), horses (5), and humans (6, 7). In some cases, *Arcanobacterium* infections have been directly linked to diseases such as pharyngitis, bacteremia, and sepsis in humans (Arcanobacterium haemolyticum) (8, 9), bovine mastitis (*A. pluranimalium*) (10, 11), and dermatitis in mink (*A. phocae*) (12).

The strain described in this study, *Arcanobacterium* sp. strain 2701, was originally isolated from an anal swab of a dead male harbor seal from the Danish island of Rømø as part of a national monitoring program in 2004. Affiliation of the strain with the genus *Arcanobacterium* was confirmed by Gram staining, CAMP-like hemolysis, and matrix-assisted laser desorption ionization–time of flight mass spectrometry (MALDI-TOF MS; Bruker Biotyper), as described previously (3, 13–15). The strain was subjected to whole-genome sequencing for phylogenetic classification.

The strain was cultivated for 48 h at 37°C under microaerobic conditions on sheep blood agar. Genomic DNA was extracted using the MagMAX Microbiome Ultra nucleic acid isolation kit (Thermo Fisher Scientific, Darmstadt, Germany) and sequenced using Illumina (San Diego, CA, USA) and Oxford Nanopore Technologies (ONT, Oxford, UK) sequencers.

An Illumina sequencing library was prepared using the Nextera DNA Flex kit. Sequencing was performed in 2 × 151-bp cycles on an Illumina NextSeq 500 sequencer using the NextSeq 500/550 midoutput kit v2.5. Trimming of short-reads using fastp v0.19.5 (16) resulted in 1.2 million high-quality paired-end reads (≥87.8% Q30).

A MinION sequencing library was prepared using the rapid barcoding kit (ONT) and sequenced on an ONT MinION sequencer, connected to an ONT MinIT v19.12.5 device (including Guppy base caller v3.2.10) using a FLO-MIN106 R9 flow cell. The resulting reads were trimmed using Porechop v0.2.3 (https://github.com/rrwick/Porechop) and quality checked using NanoStat v1.2.1 (17). In total, 28,240 reads with a read length *N*_50_ value of 8,361 bp and a mean read quality score of 11.3 were available for further analysis.

Both data sets were *de novo* assembled and circularized using Unicycler v0.4.4 including Pilon (18–20). The assembly resulted in a closed chromosome of 1,941,174 bp with a G+C content of 49%. The start point was manually set to the *dnaA* gene. For all applied software, default parameters were used.

**FIG 1:**
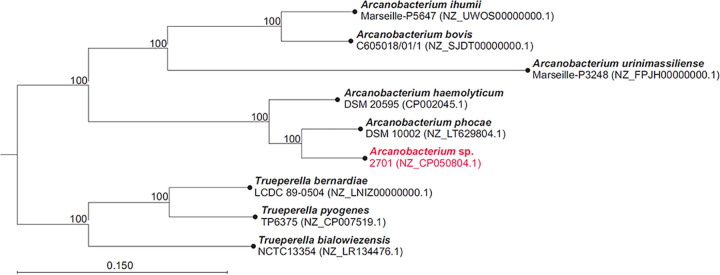
Best-scoring maximum-likelihood tree based on the comparison of the amino acid sequences of 107 essential single-copy core genes of *Arcanobacterium* sp. strain 2701, other published *Arcanobacterium* spp., and closely related *Trueperella* spp. with bcgTree. Numbers at the nodes designate bootstrap support values resulting from 100 bootstrap replicates.

The genome was deposited in the NCBI nucleotide database and annotated using the Prokaryotic Genome Annotation Pipeline v4.11 (https://www.ncbi.nlm.nih.gov/genome/annotation_prok/) (21). *Arcanobacterium* sp. strain 2701 was compared to previously published *Arcanobacterium* sp. and *Trueperella* sp. genomes locally annotated using Prokka v1.1.3 (https://github.com/tseemann/prokka). Phylogeny was inferred through amino acid sequence comparison of 107 single-copy core genes with bcgTree v1.1.0 (22–24), which uses the hmmsearch tool HMMER v3.1b1 (25) for homology identification, MUSCLE v3.8.31 (26) for multiple sequence alignment, Gblocks v0.91b (27) for selection of conserved blocks, and RAxML v8.2.4 (17) for phylogenetic analysis. Bootstrapping was performed in 100 replicates. The resulting maximum-likelihood tree was visualized in CLC Genomics Workbench v9.5.2 (Qiagen, Hilden, Germany), manually rooted using the *Trueperella* sp. node as the outgroup, and further rendered in CorelDraw v13 (Corel Corporation, Ottawa, Canada). The final tree (Fig. 1) reveals that *Arcanobacterium* sp. strain 2701 is most closely related to *A. phocae* DSM 10002.

With this sequence, we provide a high-quality reference for this potentially novel species of the genus *Arcanobacterium* that needs further analysis.

### Data availability.

The complete genome sequence has been deposited in NCBI GenBank under the accession number NZ_CP050804.1. The MinION and Illumina sequencing data were deposited in the NCBI Sequence Read Archive (SRA) under the accession numbers SRX8129176 and SRX8130151, respectively.
